# All-digital aliasing-free PWM transmitter with reduced filtering requirements

**DOI:** 10.1038/s41598-026-44436-1

**Published:** 2026-03-17

**Authors:** Muhammad Fahim Ul Haque, Hameeza Ahmed, Ted Johansson

**Affiliations:** 1 Department of Telecommunications Engineering, NED University of Engineering and Technology, Karachi, 75270 Pakistan; 2 Neurocomputation Lab, National Center of Artificial Intelligence, NED University of Engineering and Technology, Karachi, 75270 Pakistan; 3 Department of Computer Information and System Engineering, NED University of Engineering and Technology, Karachi, 75270 Pakistan; 4 Department of Electrical Engineering, Uppsala University, Uppsala, 75105 Sweden

**Keywords:** Energy science and technology, Engineering

## Abstract

The paper presents an All-Digital Aliasing-Free PWM (AF-PWM) transmitter, which combines multiphase band-limited PWM (MP-BLPWM) and accumulated *N* phase-shift pulse modulation (AN-PSPM), and its FPGA-implementation. As the architecture is based on MP-BLPWM, which generates finite harmonics PWM, this eliminates image and aliasing distortion, and improves spectral performance. However, finite harmonics PWM leads to large amplitude variation, which is converted to two voltage level signals using AN-PSPM, leading toward all-digital implementation. The transmitter’s performance is experimentally validated for 5G-NR and LTE signals, both with and without a switched-mode power amplifier (Class-D PA). Measurement results demonstrate that, when used with the PA, the transmitter achieves ACLR values of 37.9 dBc for 5G-NR and 42.2 dBc for LTE signals. Furthermore, the EVM of the proposed transmitter with the Class-D PA is measured at 1.3% for 5G-NR and 0.9% for LTE, highlighting its effectiveness for advanced wireless communication applications.

## Introduction

The Software Defined Radio (SDR)^1,2^ represents a paradigm shift in wireless communication system design, where components traditionally implemented as fixed hardware—such as mixers, filters, amplifiers, modulators, and demodulators—are instead executed through software. This architectural transformation decouples hardware from functionality, enabling unprecedented flexibility in radio system operation and reconfiguration.

In essence, an SDR system consists of a configurable radio frequency (RF) front end combined with digital signal processing (DSP) capabilities implemented on programmable hardware such as Field-Programmable Gate Arrays (FPGAs) or embedded systems^3^. The SDR system provides flexibility, quick implementation, and standard upgrades for wireless transceivers. An SDR transmitter consists of an FPGA, followed by data converters, mixers, and power amplifiers (PA). For modern wireless standards like 4G, 5G, WLAN, etc., which have high peak-to-average power ratio (PAPR), the SDR requires high-resolution data converters and linear PAs operating at large back-off to improve bandwidth efficiency.

**Fig. 1:**
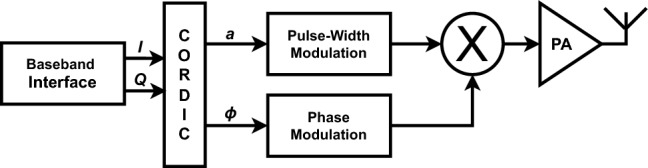
The polar pulse-width modulation transmitter (PPWMT) architecture.

All-Digital Transmitters (ADT) have entire digital transmission chains except for final filtering, which makes them ideal candidates for SDR as they provide high reconfigurability and improved power efficiency. The predominant architecture employs polar pulse-width modulation (PPWM)^4–7^, as shown in Fig. 1.

In this approach, the baseband amplitude information is converted into a pulse-width modulated (PWM) signal, with the pulse-repetition frequency (PRF) set to the intermediate frequency (IF). Simultaneously, the baseband phase information is used to generate a phase-modulated carrier at the desired transmission frequency. The PWM signal is then mixed with the phase-modulated carrier, subsequently amplified by a PA, and filtered to produce the desired transmission signal. The absence of a feedback path and the use of a single upconverted PWM path in the PPWMT result in reduced implementation complexity and lower cost. Additionally, PPWM transmitters operate the PA at either peak or zero power, a so called switched-mode PA (SMPA), resulting in high power efficiency. The advantages of lower cost, less complex implementation, and high efficiency make PPWMT the preferred choice for ADT. However, PPWM transmitters (PPWMTs) are susceptible to image distortion^8^, and their digital implementations can introduce aliasing distortion. These effects collectively degrade the adjacent channel leakage ratio (ACLR) and worsen the error vector magnitude (EVM)^8,9^, which are important specifications in different wireless communication standards.

The major techniques for reducing aliasing and imaging distortion are the aliasing-compensated PWM transmitter (AC-PWMT)^10,11^, the aliasing-free PWM transmitter (AF-PWMT)^9,12^, and its variants^13–16^.

The AC-PWMT eliminates image and aliasing distortion by quantizing the baseband signal based on PWM, sampling, and carrier frequency. The error generated due to the quantization of the baseband signal is mitigated through outphasing^10^. The additional advantage of this technique is its all-digital implementation on FPGAs^11^. At the same time, this transmitter requires two FPGA transceivers and a combiner, which increases the complexity and cost. Moreover, ACLR is slightly deteriorated due to out-of-band image distortion, aliasing distortion, and outphasing mismatch.

The AF-PWMT eliminates the aliasing and imaging distortion using finite harmonics PWM, which results in an improved ACLR and EVM, but leads to large amplitude variation instead of two voltage levels^9^. Hence, this architecture requires a high-resolution digital-to-analog converter (DAC) and a highly linear amplifier. The major variants to reduce the DAC resolution and allow nonlinearity in PAs are the Direct Digital Synthesis AF-PWMT transmitter (DDS-AFPWMT)^13,14^ and Gibbs-Phenomenon-Reduced PWM transmitter (GR-PWMT)^15–18^.

The DDS-AFPWMT implements AF-PWM signal generation using direct digital synthesis at a high sample rate. This technique’s major advantage is that it allows a low-resolution DAC and nonlinearities in the PA^13,14^, but does not allow an all-digital implementation of AF-PWMT or the use of SMPAs.

The GR-PWMT uses special filtering on finite harmonics PWM to reduce Gibbs ripple, which allows PA nonlinearities without a significant decrease in ACLR and EVM^15^. The Direct Digital Synthesis of GR-PWMT (DDS-GPT)^16^ allows further PA nonlinearities and reduces the requirements on the DAC resolution using direct digital synthesis along with dithering at a high sampling rate. Nonetheless, an all digital implementation of this technique is not possible.

In this paper, we propose an all-digital aliasing-free PWM transmitter (ADAF-PWMT) architecture and its FPGA implementation. To the authors’ knowledge, this is the first aliasing-free PWM-based transmitter (AF-PWMT) implemented on an FPGA. The ADAF-PWMT architecture combines multi-phase band-limited PWM (MP-BLPWM) with accumulated *N* phase-shift pulse modulation (AN-PSPM)^19^ to generate a two-level output signal directly from the FPGA transceiver. The signal can then be amplified using an SMPA and filtered to produce the final amplified transmit signal.

## Concept

The aliasing-free PWM and its variants show better ACLR and EVM than conventional PWM transmitters as they do not suffer from aliasing distortion. This type of transmitter does not have a two-level signal and hence cannot be implemented on FPGAs. The proposed architecture combines MP-BLPWM and AN-PSPM. The MP-BLPWM generates an amplitude-varying PWM signal, which is converted to a two-level signal using AN-PSPM, allowing an FPGA implementation. The reason to select AN-PSPM is that it provides more amplitude and phase combinations than conventional Radio-Frequency PWM (RF-PWM) for the same level of time resolution.

**Fig. 2:**
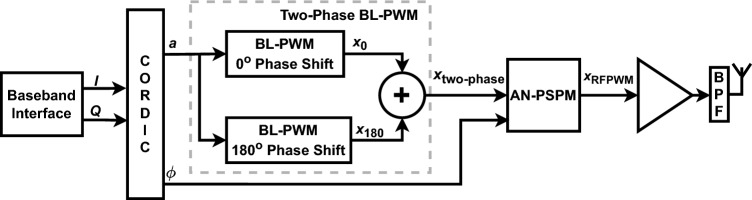
The proposed all-digital aliasing-free PWM transmitter (ADAF-PWMT) architecture.

The block diagram of the proposed ADAF-PWMT is shown in Fig. 2. The baseband *I* and *Q* signals are converted into amplitude, *a*, and phase, $\phi$, signals using a CORDIC (coordinate rotation digital computer)^20^. The amplitude signal will generate *M* phase-shifted BL-PWM signals using BL-PWM generators^21^. In our case, we are using two-phase BL-PWM, so two $180^{\circ }$ phase-shifted BL-PWM signals are generated, which are mathematically expressed as:

1a $$\begin{aligned} x_{0}(t) =a(t)+\frac{2}{\pi }\sin \left( \pi a(t)\right) \cos \left( \omega _\text {IF}t\right) +\sum _{k=2}^{K}\frac{2}{k\pi }\sin \left( k\pi a(t)\right) \cos \left( k \omega _\text {IF}t\right) \end{aligned}$$

1b $$\begin{aligned} x_{180}(t) =a(t)-\frac{2}{\pi }\sin \left( \pi a(t)\right) \cos \left( \omega _\text {IF}t\right) +\sum _{k=2}^{K}\frac{2}{k\pi }\left( -1\right) ^k\sin \left( k\pi a(t)\right) \cos \left( k \omega _\text {IF}t\right) \end{aligned}$$

where *a*(*t*) is is the amplitude, *k* is the harmonic number, *K* is the total number of harmonic component in the BL-PWM signal, and $\omega _\text {IF}$ is the PRF of the BL-PWM signal. The selection of PRF is dependent on the final filtering. Higher PRF leads to relaxed final filtering, while lower PRF leads to stringent filtering. The minimum PRF is limited by baseband signal bandwidth and its sampling rate.

The *M* phase-shifted BL-PWM signals are added to generate the MP-BLPWM. The advantage of MP-BLPWM is the filter relaxation, at the cost of considerable amplitude variation. In our case, two $180^{\circ }$phase-shifted signals are added to generate a two-phase BL-PWM signal, which is mathematically expressed as:

2 $$\begin{aligned} x_\text {two-phase}=2a(t)+\sum _{k=1}^{K}\frac{2}{k\pi }\sin \left( 2k\pi a(t)\right) \cos \left( 2k\omega _\text {IF}t\right) . \end{aligned}$$

**Fig. 3:**
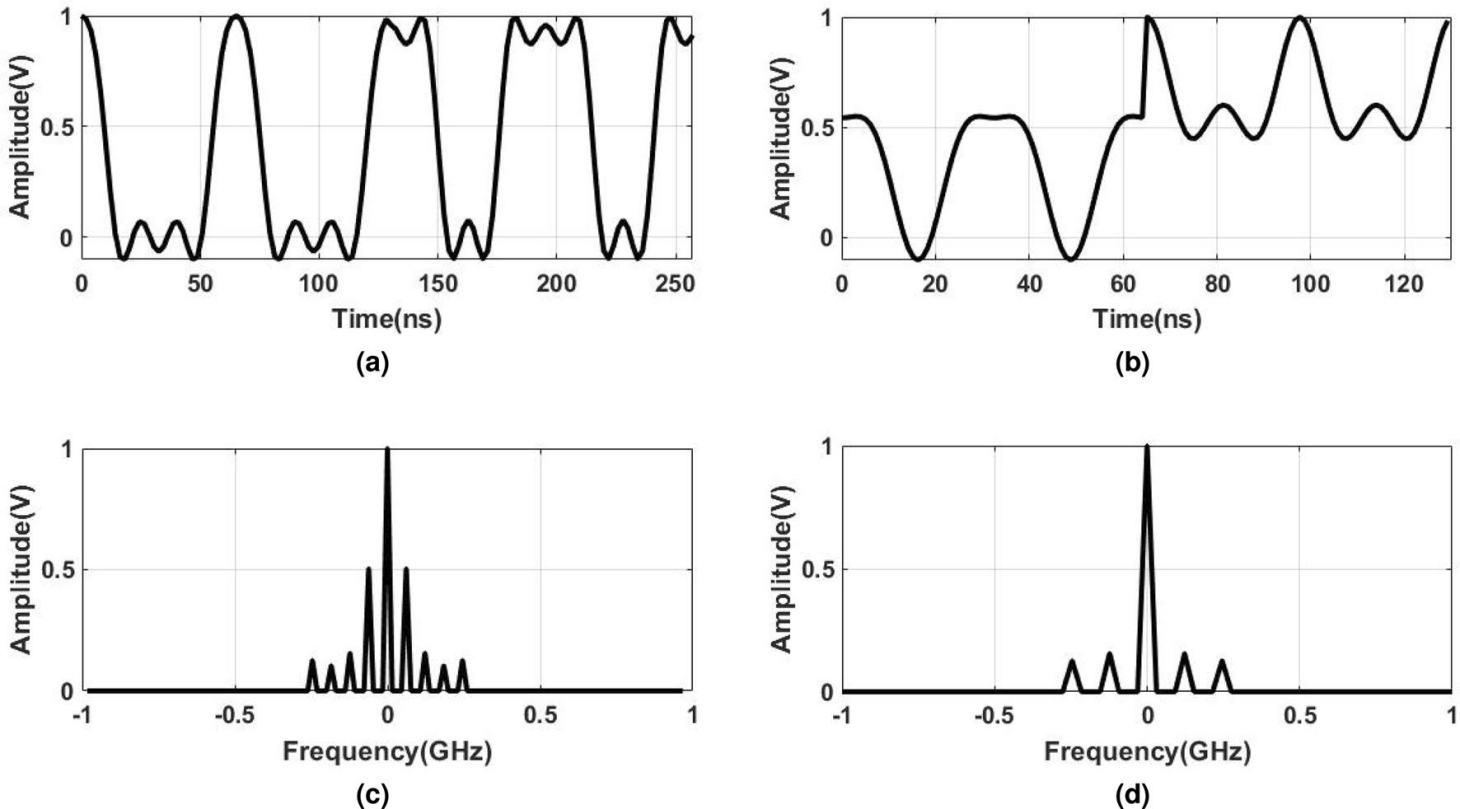
Time-domain signal and spectrum of BL-PWM and two-phase BL-PWM.

The frequency and time-domain comparisons of BL-PWM and two-phase BL-PWM are shown in Fig. 3a–d. It is evident that the two-phase BL-PWM has harmonic cancellation at an odd multiple of the PRF, in contrast to the BL-PWM that shows harmonics at multiples of the PRF. However, two-phase BL-PWM has significant amplitude variations compared to BL-PWM. The variations in the BL-PWM and MP-BLPWM do not allow all digital implementations. In the ADAF-PWMT, the MP-BLPWM amplitude is translated to two voltage levels using AN-PSPM, hence allowing an all-digital implementation.

The AN-PSPM block generates RF pulses based on MP-BLPWM amplitude and baseband phase signals using the accumulation of phase-shifted pulse trains with a duty cycle $T_\text {res}/T_\text {c}$, where $T_\text {res}$ is the minimum pulse width of the pulse train, and $T_\text {c}$ is the period of the pulse train, equal to the carrier period of the proposed transmitter. For FPGAs, $T_\text {res}$ is determined by the speed of the FPGA transceiver. Notably, the $T_\text {res}/T_\text {c}$ directly influences the RF performance of the transmitter; higher ratios yield improved ACLR and EVM by enhancing amplitude and phase resolution. AN-PSPM generates a larger set of RF pulses that are not necessarily continuous, in contrast to RFPWM, which has a smaller set of RF pulses of only continuous width. As a result, AN-PSPM can map a larger combination of amplitudes and phases compared to RFPWM.

The mathematical form of the AN-PSPM for the ML-PWM amplitude and baseband phase is given in (3a), where $\tau _\text {min}$ is the minimum resolution of the pulse width, $T_\text {c}$ is the carrier time period, $\psi _q$ is the phase shift of the pulse train, *Q* is the total number of pulses accumulated, and $\delta _q$ is 1 or 0 based on the unique combination of amplitude and phase. Moreover, in (3a) $A_1$ is the amplitude of the ML-PWM signal $(x_\text {two-phase})$ and $\Phi _1$ is the baseband phase $(\phi )$, which determines the value of $\delta _\text {q}$. Finally, $A_n$ and $\phi _n$ denote the amplitude and phase of the higher-order harmonics, which are subsequently removed by filtering after power amplification.

3a $$\begin{aligned} {X}_\text {RFPWM}(t) = \sum _{q=1}^Q\frac{\delta _q\tau _\text {min}}{T_\text {c}} + A_1\cos (\omega _\text {c} t + \Phi _{1})+\sum _{n=2}^\infty \frac{2}{n\pi } A_n\cos (n\omega _\text {c} t+ n\Phi _{n}) \end{aligned}$$

where

3b $$\begin{aligned} \begin{aligned} A_1=\sin \left( \frac{\pi \tau _\text {min}}{T_\text {c}}\right) \sqrt{\left[ \sum _{q=1}^Q\delta _q\cos (\psi _q)\right] ^2 + \left[ \sum _{q=1}^Q\delta _q\sin (\psi _q)\right] ^2 } \end{aligned} \end{aligned}$$

3c $$\begin{aligned} \begin{aligned} A_n=\sin \left( \frac{n\pi \tau _\text {min}}{T_\text {c}}\right) \sqrt{\left[ \sum _{q=1}^Q\delta _q\cos (n\psi _q)\right] ^2+ \left[ \sum _{q=1}^Q\delta _q\sin (n\psi _q)\right] ^2} \end{aligned} \end{aligned}$$

3d $$\begin{aligned} \Phi _{1}=\arctan \left[ \frac{\sum _{q=1}^Q\sin (\psi _q)}{\sum _{q=1}^Q\cos (\psi _q)}\right] \end{aligned}$$

3e $$\begin{aligned} \Phi _{n}=\arctan \left[ \frac{\sum _{q=1}^Q\sin (k\psi _q)}{\sum _{q=1}^Q\cos (k\psi _q)}\right] \end{aligned}$$

**Fig. 4:**
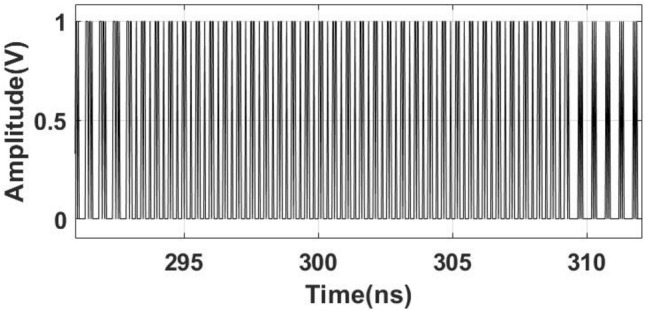
Time-domain signal of two-phase BL-PWM after AN-PSPM encoding.

Figure 4 shows the resultant time-domain AN-PSPM signal for two-phase BL-PWM at a baseband phase of $0^{\circ }$. The figure clearly shows that AN-PSPM converts two-phase BL-PWM into two voltage-level signals, which makes an all-digital implementation of aliasing-free PWM on FPGA possible. The AN-PSPM signal is then amplified by an SMPA and finally band-pass filtered to generate the amplified transmit signal.

**Fig. 5:**
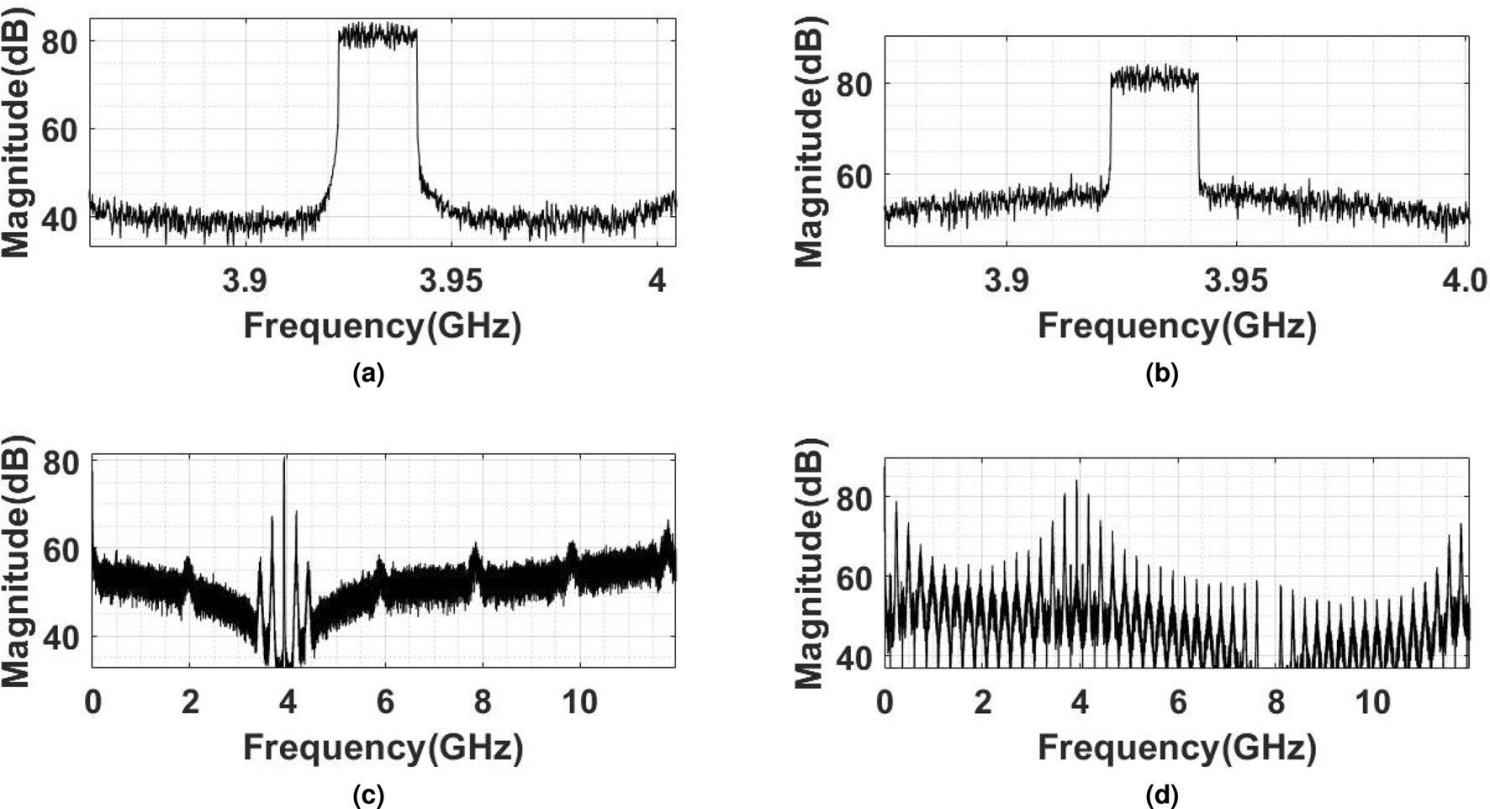
Narrowband and wideband simulated spectra of ADAF-PWMT and PPWMT: (a) narrowband spectrum of ADAF-PWMT, (b) narrowband spectrum of PPWMT, (c) wideband spectrum of ADAF-PWMT, (d) wideband spectrum of PPWMT.

The comparison of the narrowband and wideband simulated spectra of the proposed ADAF-PWMT transmitter and a PPWMT for a 20 MHz 5G-NR signal is shown in Fig. 5a–d. The carrier signal for both transmitters is 3.92 GHz. It is apparent from Fig. 5a and b that the ADAF-PWMT has better spectral performance on the adjacent channels than the PPWMT, which is attributed to the absence of image distortion, achieved by using a finite harmonics band-limited PWM in the ADAF-PWMT. The PPWMT employs infinite harmonics baseband PWM. This results in the repetition of the infinite baseband PWM spectrum at multiple carrier frequencies due to the square wave carrier. As a result, imaging harmonics appear on the desired transmit signal. In contrast, ADAF-PWMT uses BL-PWM, which has a finite spectrum. This prevents image distortion from the repetition of the BL-PWM spectrum at multiples of the PRF of the carrier square wave. These phenomena are clearly evident from Fig. 5c and d. Additionally, the ADAF-PWMT exhibits significantly lower power harmonics near the transmit signal compared to the conventional polar PWM transmitter, which is due to the use of multiphase BL-PWM. Moreover, the harmonics at the ADAF-PWMT output can be eliminated by increasing the count of phase-shifted BL-PWM signals.

## Implementation

**Fig. 6:**
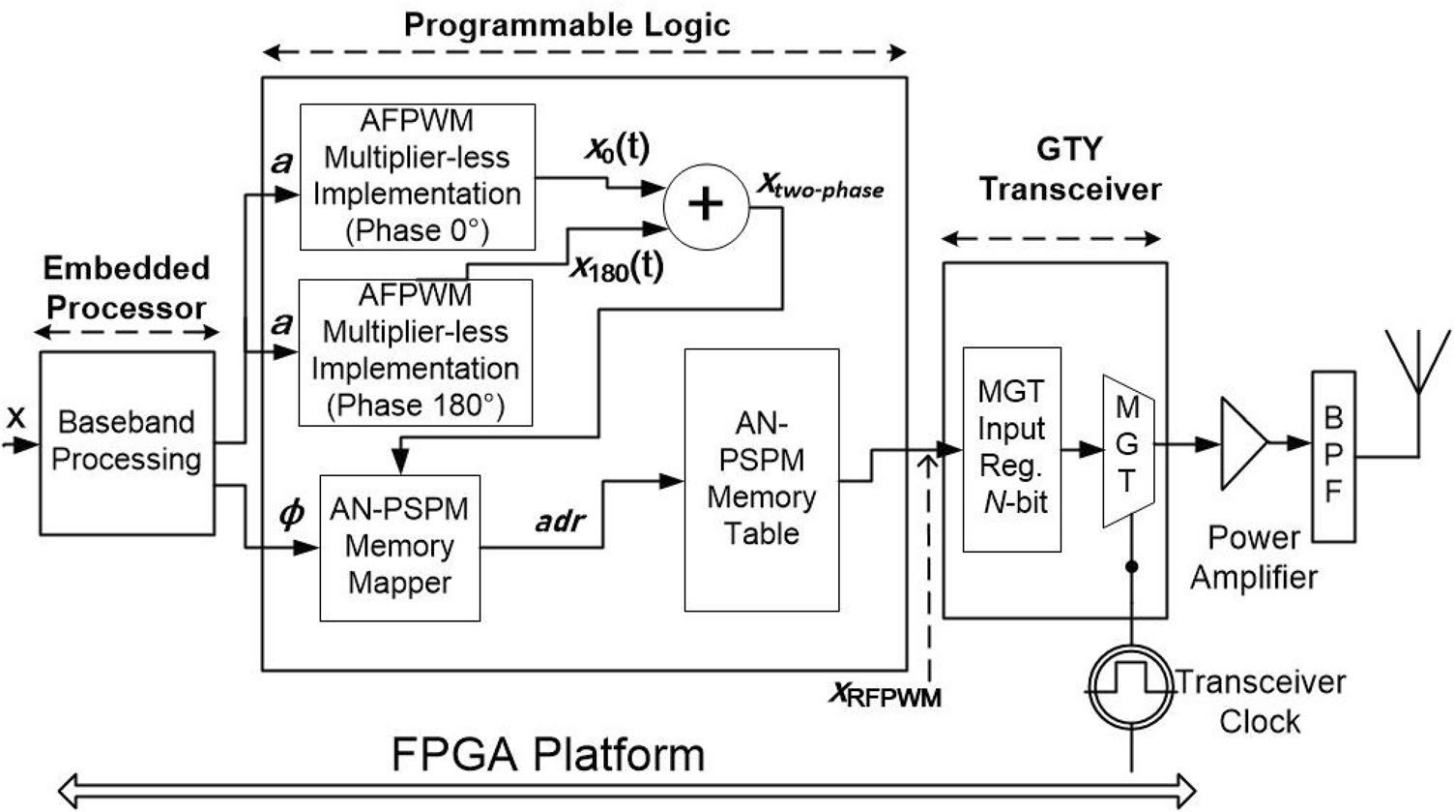
FPGA-implementation of the ADAF-PWMT.

Implementation of the proposed ADAF-PWMT architecture has been carried out on a Zynq UltraScale+ RFSoC ZCU111 Evaluation Kit. This FPGA has GTY hard IP blocks that support up to 28.2 Gbps line rate, as well as configurable input/latching register widths that ranges from 32 bits to 128 bits. The implementation of the ADAF-PWMT is also possible on low-cost FPGAs with a transceiver rate of 16 Gbps, however, this may result in a lower maximum carrier frequency or a reduced ACLR. A simplified block diagram of the implementation is depicted in Fig. 6, which uses the stored memory concept with pre-calculated values for the BL-PWM pulses and AN-PSPM pulses.

The baseband processing receives an input signal in *I*/*Q* format. This processing block converts the signal into polar format, producing the amplitude, *a*, and the phase signal, $\phi$. The amplitude signal generates the two $180^{\circ }$ phase-shifted BL-PWM signals using multiplier-less lookup table (LUT) based implementation^21^. These signals are added to generate a two-phase BL-PWM signal.

Similarly, the set of AN-PSPM pulses has also been pre-built from the software and stored in the FPGA on-chip memory (Block RAM). There is a mapper logic required in the system to correctly identify the AN-PSPM pulse from the input phase signal and corresponding two-phase BL-PWM output. This mapping logic is implemented in the AN-PSPM Memory mapper block, which generates the final address of the appropriate PWM pulse data stored in the AN-PSPM memory. Finally, the pulse is sent to the transceiver block, which captures the parallel *N*-bit pulses and serializes them at the desired rate, hence achieving the overall concept formulated in Fig. 2. It is possible to generate multiple modulation bandwidths and carrier frequencies while keeping the AN-PSPM memory constant. In this case, bandwidth can be changed through the PRF of the BL-PWM and carrier frequency by changing the transceiver rate. The FPGA resource utilized by the proposed transmitter implementation is listed in Table 1.

Also the PPWMT transmitter was implemented on the FPGA and were used as a comparison in the measurements, as reported in the next section.

**Table 1:** FPGA resource utilization for the ADAF-PWMT implementation.

Resource	Used	Available	Utilization (%)
CLB LUTs	2388	425280	0.56
CLB Registers	2640	850560	0.31
Block Rams (RAMB36E)	174	1080	16.0
GTYE4_CHANNEL	1	16	-
GTYE4_COMMON	1	4	-

## Measurement results

The performance of the ADAF-PWMT was evaluated using both 5G NR and LTE signals at carrier frequencies of 720 MHz and 1.75 GHz at multiple BWs. The signals were generated in Keysight System Vue, re-formatted in MATLAB and input to the Ultrascale+ RFSoC ZCU111 Evaluation board, which implements the ADAF-PWMT. For the 720 MHz carrier, this signal was amplified by an SMPA, with its output connected to the spectrum analyzer.

**Fig. 7:**
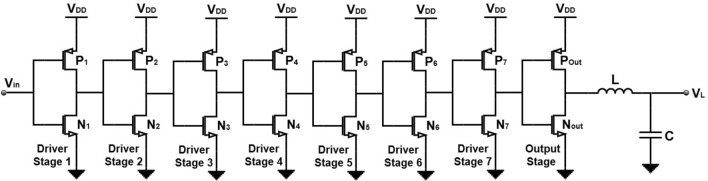
Schematic diagram of the class-D PA.

**Fig. 8:**
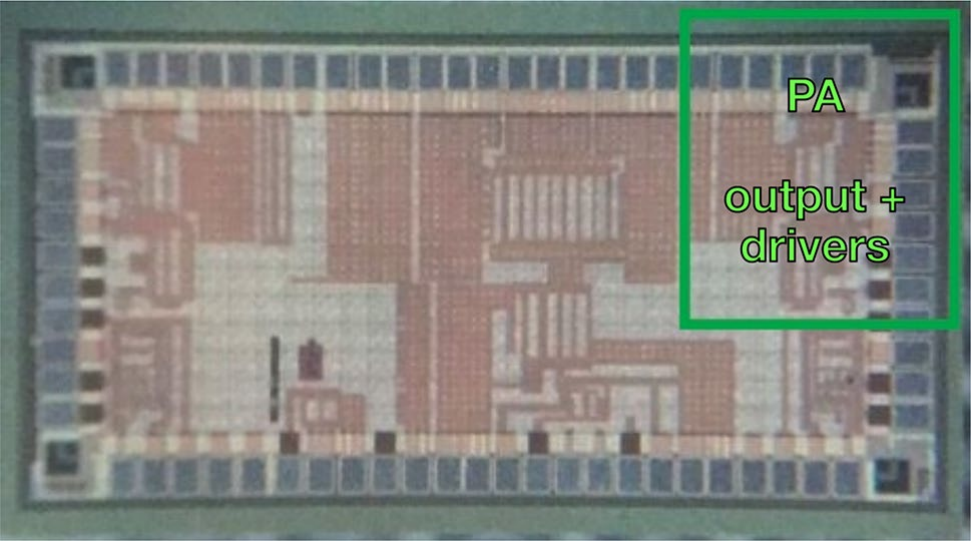
Chip micrograph. The size of the total chip is 2 x 1 $\hbox {mm}^2$. The class-D PA used in this work is marked with a green box. The area of the PA is 0.3 $\hbox {mm}^2$.

The SMPA^22^ is a 130 nm CMOS class-D PA using thick-oxide transistors operating at 2.5 V, and was implemented using cascaded driver inverter chains as shown in Fig. 7, with NMOS/PMOS gate widths ranging from 3.3/6.6 um for the first stage to 7200/14400 um for the output stage, with the chip photo shown in Fig. 8. The matching network of the output stage is an L-type implementation on a PCB, utilizing a strip line and a discrete capacitor.

**Fig. 9:**
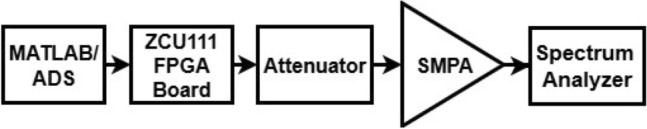
Block diagram of the measurement setup.

**Fig. 10:**
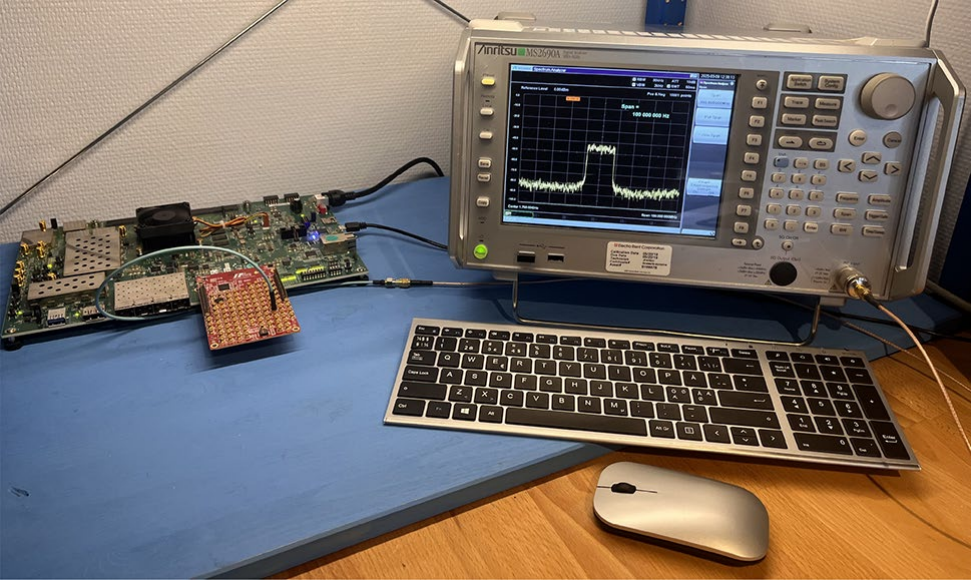
Lab measurement setup.

For the 1.75 GHz carrier, the signal from the transceiver was directly fed to the spectrum analyzer without amplification. The block diagram and photo of the lab measurement setup are shown in Figs. 9 and 10, respectively.

**Fig. 11:**
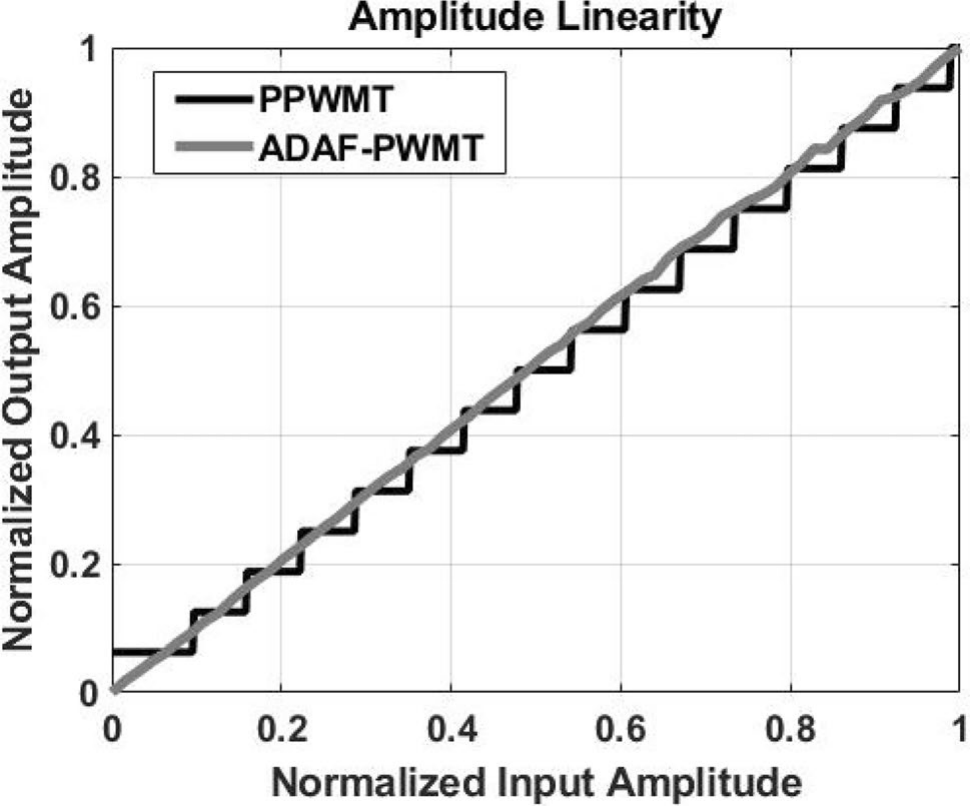
Measured linearity of ADAF-PWMT and PPWMT.

The amplitude linearity (AM-AM) comparison between the ADAF-PWMT and the PPWMT at 720 MHz carrier is depicted in Fig. 11, where the normalized output amplitude is plotted against the normalized input amplitude. The ADAF-PWMT exhibits a linear input-output relationship, whereas the PPWMT demonstrates a staircase characteristic due to imaging and aliasing distortion. Simulations indicate that the ADAF-PWMT achieves an effective resolution equivalent to a 12-bit DAC, with both INL and DNL maintained below 0.5 LSB, while the PPWMT demonstrates an effective resolution comparable to an 8-bit DAC under the same INL and DNL constraints for a 720 MHz carrier frequency and a 11.52 GS/sec transceiver sampling rate. For both transmitter architectures, the effective DAC resolution increases with the ratio of the transceiver sampling frequency to the carrier frequency.

**Fig. 12:**
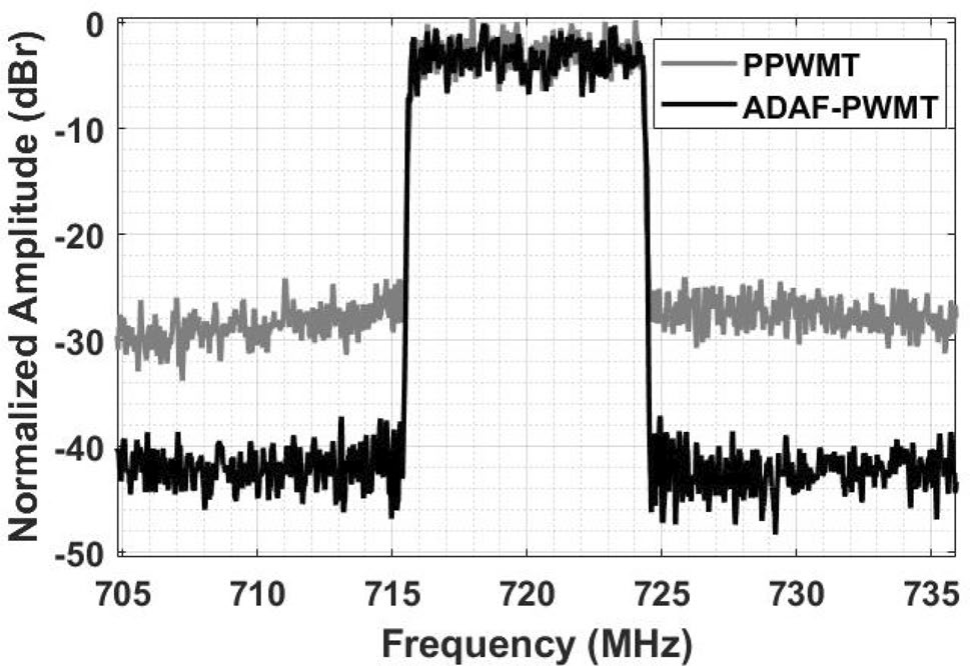
Measured spectra of ADAF-PWMT and PPWMT at the output of the SMPA.

The normalized output spectra of the ADAF-PWMT and the PPWMT for a 10 MHz 5G-NR signal at 720 MHz carrier are shown in Fig. 12. A low noise floor and a high dynamic range compared to PPWMT is clearly observed. The degraded spectral performance of PPWMT is due to aliasing and imaging distortion.

**Fig. 13:**
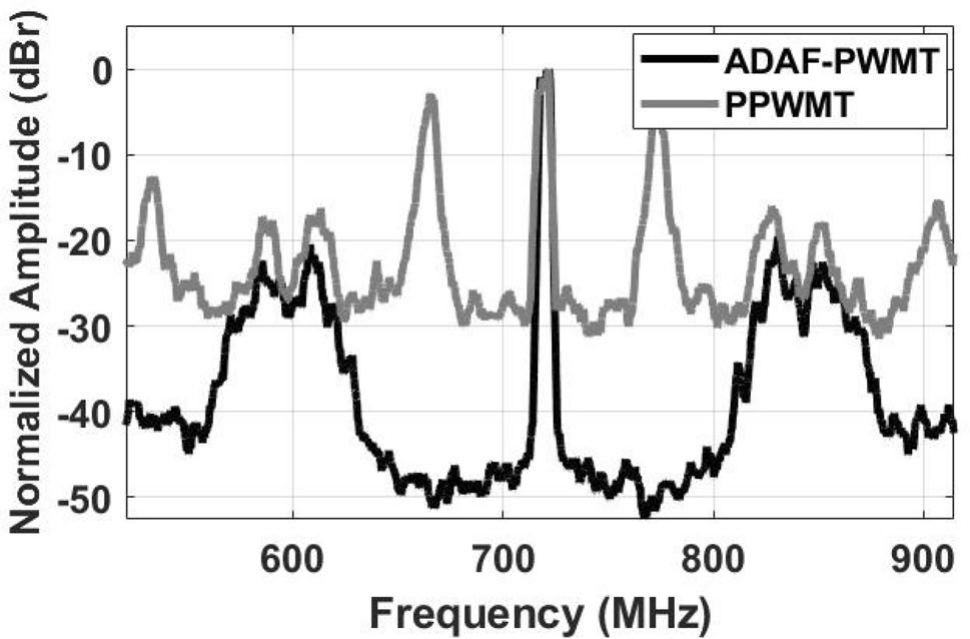
Measured extended spectra of ADAF-PWMT and PPWMT.

Figure 13 illustrates the extended output spectra of the ADAF-PWMT and the PPWMT for a 10 MHz 5G-NR signal at a 720 MHz carrier frequency, evaluated without an SMPA. For a fair comparison, the PRF of the BL-PWM signal in the ADAF-PWMT and the PWM signal in the PPWMT is set to 60 MHz. As shown in the figure, the first harmonic of the PPWMT is 60 MHz away from the desired transmit signal, corresponding directly to the PWM PRF. In contrast, the first harmonic of the ADAF-PWMT is 120 MHz away from the desired transmit frequency, i.e., twice the PRF of the BL-PWM signal. Moreover, the amplitude of the first harmonic is significantly lower than that of the PPWMT. These improvements are attributed to the two-phase BL-PWM operation employed in the proposed architecture. As the number of phase-shifted BL-PWM signals increases, the first harmonic is pushed farther away from the desired transmit signal while its amplitude is reduced, resulting in improved spectral performance.

## Discussion

In the last decade, AF-PWMT^13,14,23^ and GR-PWMT^15–18^ architectures have been studied extensively. Using DACs with a large number of bits, ACLRs from 35 dBc to 52 dBc for signals, with PAPR ranging from 5 dB to 9 dB, have been achieved^17,18,23^.

Architectures focusing on DACs with reduced number of bits have been presented^13,14,16^. The Direct Digital Synthesis GR-PWMT (DDS-GPT)^16^ shows through simulations that ACLR performance decreases as DAC bits are reduced; an ACLR of 55 dBc with a 10-bit DAC is reduced to 38 dBc for a 2-bit DAC.

The transmitter in this work is an All-Digital AF-PWMT (ADAF-PWMT) with no DAC. Table 2 summarizes the measured performance of the ADAF-PWMT in comparison with state-of-the-art AFPWMT and GRPWMT architectures employing low-resolution DACs, as well as with conventional AFPWMT and fully digital PPWMT implementations. Unlike low-resolution DAC-based AFPWMT architectures. which rely on two linear PAs and an RF combiner (first three columns of Table 2), the ADAF-PWMT employs a single Class-D PA and eliminates the need for a DAC. For a high-PAPR 5G-NR waveform, the ADAF-PWMT achieves an ACLR of 37.9 dBc, outperforming the 31.1 dBc and 31.9 dBc reported for the DDS-AFPWMT and DDS-GPT architectures, respectively. In addition, it attains a superior EVM of 1.3%, compared to 5.3% and 2.1% for the DDS-GPT and DDS-AFPWMT, respectively.

When compared to the PPWMT, the ADAF-PWMT achieves a significantly improved ACLR of 42.1 dBc versus 24.2 dBc, while maintaining similar power efficiency for an LTE signal, and further demonstrates a substantially lower EVM of 0.9% compared to 19.1% for the PPWMT. In comparison with conventional AFPWMT, the first spectral harmonic of the ADAF-PWMT is located 120 MHz away from the desired signal at 720 MHz, corresponding to twice the BL-PWM PRF, whereas the first harmonic of the AFPWMT appears only 40 MHz away from the desired signal at 900 MHz, equal to the BL-PWM PRF. This increased harmonic spacing is enabled by the multiphase BL-PWM operation of the proposed architecture.

As further shown in Table 2, increasing the BL-PWM PRF from 36 MHz to 60 MHz and 120 MHz results in harmonic spacings of 72 MHz, 120 MHz, and 240 MHz from the desired signal (columns 7, 6, and 8), demonstrating that the harmonic spacing scales directly with the BL-PWM PRF.

Finally, the ADAF-PWMT exhibits lower average efficiency than its peak efficiency due to the varying duty cycle of the AN-PSPM modulation, as Class-D PAs achieve maximum efficiency at 50% duty cycle and degrade in efficiency at lower duty cycles. If using a ZVS Contour SMPA, which maintains efficiency at reduced duty cycles, this would increase the average efficiency of the ADAF-PWMT.

In summary, the ADAF-PWMT maintains better ACLR and EVM compared to prior architectures at a larger bandwidth of 20 MHz and higher carrier frequency of 1.75 GHz at the cost of higher transceiver rate of FPGA. This performance is achieved with an all-digital FPGA implementation, which is, as per the author’s knowledge, the first complete aliasing-free transmitter implementation on an FPGA.

**Table 2:** Measured performance comparison of the proposed ADAF-PWMT with state-of-the-art low-resolution DAC-based AF-PWMT architectures, the conventional AF-PWM transmitter, and the all-digital PPWMT.

	**DDS**^16^	**DDS**^16^	**DDS**^13^	**PPWMT**^11^	AF-PWMT^9^	ADAF	ADAF	ADAF
	GPT	GPT.	AFPWMT			PWMT	PWMT	PWMT
						This Work	This Work	This Work
Result Type	Sim.	Meas.	Meas.	Meas.	Meas.	Meas.	Meas.	Meas.
Standard	5G-NR	5G-NR	5G-NR	LTE	DMT	5G-NR	LTE	5G-NR
BW (MHz)	20	20	20	20	5	10	3	20
PAPR (dB)	5.1	5.1	9.2	5.5	7.0	12	7.7	12
f_c (MHz)	-	836.5	836.5	640	900	720	720	1750
ACLR (dBc)	38	31.1	31.9	24.2	-	37.9	42.1	39.6
EVM (%)	1.3	5.3	2.1	19.1	-	1.3	0.9	1.1
DACs	2	2	2	0	2	0	0	0
DAC bits	2	2	4	N/A	16	N/A	N/A	N/A
Avg. Eff.(%)	N/A	23.4	26.9	15.5	33	10	15.4	N/A
Peak Eff.(%)	N/A	70	70	-	>72	34	34	N/A
Pout(dBm)	N/A	21.4	20.3	-	-	10.1	14.6	N/A
Peak Power (dBm)	N/A	-	29.1	27.8	39.0	22	22	N/A
PA Class	N/A	Class C	Class C	Class D	Class AB	Class D	Class D	N/A
No. of PA	N/A	2	2	1	1	1	1	N/A
PA	N/A	Discrete PA	Discrete PA	CMOS	LDMOS	CMOS	CMOS	N/A
Technology		p-HEMT	p-HEMT	130 nm	30V	130 nm	130 nm	
Combiner	1	1	1	0	0	0	0	0
Sample Rate (GS/sec)	10	10	4	10.8	-	11.7	11.7	28
All Digital FPGA Imp.	No	No	No	Yes	No	Yes	Yes	Yes
First Harmonic	Not	Not	Not	Not	40	120	72	220
Spacing (MHz)	Presented	Presented	Presented	Presented				
Peak First	Not	Not	Not	Not	Not	−16.2	−9.7	-
Harm Power(dBm)	Presented	Presented	Presented	Presented	Presented			

## Conclusion

The paper presents the concept, FPGA implementation, and experimental validation of an all-digital aliasing-free PWM transmitter (ADAF-PWMT). The proposed architecture demonstrates superior linearity and spectral performance compared to polar PWM transmitters, primarily due to its effective elimination of imaging and aliasing distortion. Additionally, in contrast to low-resolution DAC variants of AF-PWMT, the proposed solution achieves significantly improved ACLR and EVM metrics while maintaining a fully digital design on the FPGA platform. Comprehensive measurements with and without the Class-D PA confirm robust performance for 5G-NR and LTE signals. Specifically, when measured with the Class-D PA, the transmitter achieves ACLR values of 37.9 dBc for 5G-NR and 42.1 dBc for LTE, alongside EVM values of 1.3% and 0.9%, respectively. These results highlight the potential of the ADAF-PWMTfor efficient, high-performance digital wireless communication systems.

## Data Availability

The datasets generated during and/or analysed during the current study are available from the corresponding author on reasonable request.
